# Knockout of MDA-9/Syntenin (SDCBP) expression in the microenvironment dampens tumor-supporting inflammation and inhibits melanoma metastasis

**DOI:** 10.18632/oncotarget.10040

**Published:** 2016-06-21

**Authors:** Swadesh K. Das, Chunqing Guo, Anjan K. Pradhan, Praveen Bhoopathi, Sarmistha Talukdar, Xue-Ning Shen, Luni Emdad, Mark A. Subler, Jolene J. Windle, Devanand Sarkar, Xiang-Yang Wang, Paul B. Fisher

**Affiliations:** ^1^ Department of Human and Molecular Genetics, Virginia Commonwealth University, School of Medicine, Richmond, VA, USA; ^2^ VCU Institute of Molecular Medicine, Virginia Commonwealth University, School of Medicine, Richmond, VA, USA; ^3^ VCU Massey Cancer Center, Virginia Commonwealth University, School of Medicine, Richmond, VA, USA

**Keywords:** melanoma differentiation associated gene-9/syntenin (mda-9/syntenin), syndecan binding protein (SDCBP), tumor microenvironment, myeloid-derived tumor suppressor cells (MDSC), interleukin −17A (IL-17A)

## Abstract

Cancer development and progression to metastasis is a complex process, which largely depends on bidirectional communication between tumor cells and their microenvironment. *Melanoma differentiation associated gene-9* (*mda-9*, also known as *Syntenin-1*, *SDCBP*), a gene first cloned by our group, is robustly expressed in multiple cancers including melanoma and contributes to invasion and metastasis in a tumor cell-intrinsic manner. However, the role of MDA-9/Syntenin in the tumor cell-extrinsic microenvironment remains unclear even though MDA-9/Syntenin is ubiquitously expressed in most organs that are active metastatic sites for melanoma, e.g., lung, lymph node, brain, and liver. In this study, we explored the effect of environmental *mda-9/syntenin* expression on melanoma growth and metastasis using multiple immunocompetent animal models, syngeneic B16 xenograft and intravenous B16 mouse model and a genetically engineered mouse (GEM) model of melanoma. Host-deficient expression of *mda-9/syntenin* in mice negatively impacted on subcutaneously implanted B16 tumor growth and lung metastasis. Absence of MDA-9/Syntenin in the lung microenvironment suppressed tumor growth by modulating *in situ* Interleukin 17A (IL17A) expression and impaired the recruitment of myeloid derived suppressor cells (MDSCs) and Th17 cells as compared to genetically wild type animals. Additionally, loss of *mda-9/syntenin* expression in a spontaneous melanoma model (melanocyte-specific *pten* loss and *Braf*V600E mutation) significantly delayed tumor initiation and suppressed metastasis to the lymph nodes and lungs. The present study highlights a novel role of *mda-9/syntenin* in tumor-promoting inflammation and immune suppression. These observations along with other documented roles of MDA-9/Syntenin in cancer and metastasis support the potential relevance of MDA-9/Syntenin in the carcinogenic process and as a target for developing improved therapies by using either genetic or pharmacologic approaches to treat and prevent melanoma and other cancers.

## INTRODUCTION

Metastasis is a multifaceted process that initiates with the migration of tumor cells from a primary site and culminates in the formation of new secondary tumors in distant organs [1]. For successful survival and growth of metastatic cells in their new environment, disruption of homeostasis of the host organ is crucial [2]. Each organ contains multiple cell types organized in an ordered/structured architecture and numerous molecular/biochemical processes are necessary to maintain physiological functions [1]. Through mechanisms that require further clarification, specific biochemical and molecular changes, either induced by invading cancer cells or occurring as spontaneous events, can disrupt this organization and facilitate tumor cell colonization and growth [3]. Numerous studies support the importance of cross talk between the resident cells (in target organs) and invading cells in mediating metastasis [3]. In principle, the homing organ can serve as a ‘perfect ecosystem’ that can provide nutritional support and protection from the immune system to facilitate tumor growth.

Syndecan binding protein (SDCBP), *mda-9/syntenin* [4–6], a temporally expressed gene was initially cloned from terminally differentiating human melanoma cells as *mda-9/syntenin* in 1993 and described in detail in 1996 [4, 5]. Subsequently, our primary research emphasis has been to define the role of *mda-9/syntenin* in cellular transformation and metastasis. We provided definitive evidence that *mda-9/syntenin* is a pro-metastatic gene when expressed in immortal normal human cells and in human cancer cells of diverse origin with an ability to induce invasion and experimental metastasis [7–11]. The diverse roles of *mda-9/syntenin* (SDCBP) in exosome biogenesis [12–16], intracellular trafficking [17, 18], neuronal differentiation [19–21], immune cell migration [22–25] and anti-viral activity [26, 27] are also current areas of intense investigation in multiple laboratories. In total, these studies validate the functional importance of MDA-9/Syntenin in maintaining both normal cellular physiology and promoting cancer progression. Recently, Tamura *et al*. reported that *mda-9 (syntenin-1*) knockout mice exhibit abnormalities in immunoglobulin production and have a modified intestinal microbiota [28]. Current studies support the hypothesis that *mda-9/syntenin* is involved in multiple signaling cascades under both physiological and pathological conditions and these processes affect various phenotypes in a tissue/organ context-dependent manner. However, the physiological role of MDA-9/Syntenin (SDCBP) in the target organ niche remains to be explored. At present, we have a clearer appreciation of how MDA-9/Syntenin facilitates tumor cell invasion from a primary tumor site [7–11], i.e., how this protein regulates autonomous and non-autonomous signaling of tumor cells to degrade the extracellular matrix (ECM) [7–9, 29–32], promotes migration [29–31, 33], induces angiogenesis [11, 33] and facilitates escape from the primary tumor niche. Since the MDA-9/Syntenin protein is also expressed in multiple organs under physiological conditions, it is relevant to define the precise role of basal expression of this protein, if any, in the context of the host organ microenvironment, which is a critical regulator of metastasis.

Accumulating evidence suggests that a local immune-suppressive and inflammatory microenvironment is a key element for tumor progression and invasion [34–36]. Myeloid derived suppressor cells (MDSCs), a heterogeneous population of cells of myeloid origin, have garnered attention due to their immune suppressive functions in a tumor bearing host [37–39]. These effects are elicited by suppressing effector T cells [37], converting naïve CD4^+^ T cells to regulatory T cells (Tregs) [40] and inhibiting T cell trafficking [41]. CD4^+^Th17, a subset of CD4^+^ T cells, is an additional type of immune suppressive cell that also infiltrates tumors and correlates with tumor progression [42]. Interleukin 17A (IL-17A), a pro-inflammatory cytokine secreted by CD4^+^ Th17 cells, triggers tumor cells to produce interleukin 6 (IL-6), which in turn activates STAT3-dependent survival and angiogenesis [43]. Additionally, IL-17 production in the tumor microenvironment promotes infiltration of MDSCs to promote immune suppression and to amplify tumor-promoting inflammation [44]. The behavior of cancer cells is influenced to a great extent by various cytokines produced by resident immune or non-immune cells in the tumor microenvironment in response to invading tumor cells. In this study, we show that lack of *mda-9/syntenin* expression in the host lung influences the local inflammatory network, indicated by the reduced level of pro-inflammatory cytokines such as IL-6 and IL-17A, as well as diminished accumulation of Th17 cells and MDSCs. This defect in tumor-supporting inflammation strongly suppresses tumor progression as evidenced by a delay and reduction in metastatic melanoma development.

## RESULTS

### Phenotype of *mda-9/syntenin* knockout (*mda-*9) mice

**Figure 1:**
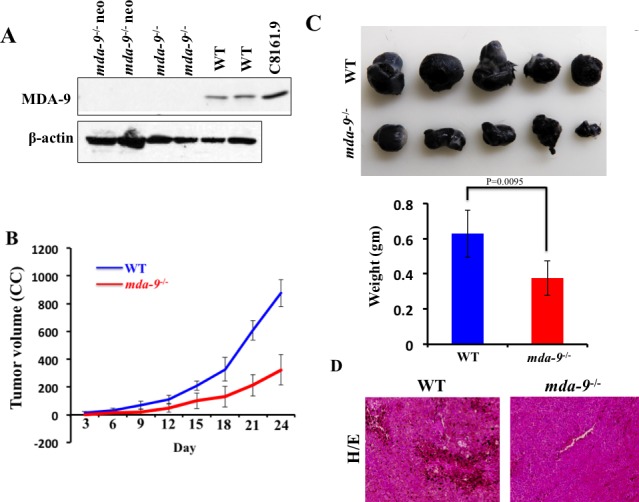
B16 xenograft tumor growth is suppressed in *mda-9/syntenin*-deficient mice **A.** MDA-9/Syntenin expression in the lungs of *mda-9* KO (*mda-9*) animals. Western blot analysis of MDA-9/Syntenin expression in lung tissue from mice homozygous for the floxed allele (CKO), the “knockout first” allele (KO neo), the final knockout allele (KO) or the wild type allele. β-actin was used as loading control. **B.** Growth kinetics of B16 xenograft tumors in experimental animals (WT and *mda-9*). Tumor volumes were calculated on every 3^rd^ day based on the calculations described in Methods and Materials. Average volumes from five mice are presented ± S.D. **C.** Photographs of tumors at the end of this study (24 days after injection). Average weight of tumors from each experimental group is presented. Data represents value from 5 mice with ± S.D. **D.** Representative photograph of hematoxylin/eosin-stained tumor sections.

As reported previously by Tamura *et al.* [28] using *syntenin-1*-deficient mice, we did not observe any phenotypic differences (Supplementary Figure S1), embryonic lethality, developmental abnormalities, or adult sterility in our *mda-9*^−/−^ mice. Additionally, body weight changes up-to six months and various organ weights at 3 months were similar between *mda-9*^−/−^ and wild type (WT) C57BL/6 mice (data not shown). We confirmed in our *mda-9*^−/−^ mice the absence of MDA-9/Syntenin protein in multiple tissues (lung shown) from mice homozygous for the knockout (*mda-9*^−/−)^ allele as well as the original “knockout first” (*mda-9*^−/−^ neo) allele (Figure 1A).

### *mda-9*-deficient mice are less supportive for murine B16-derived xenograft tumor growth

Our previous studies firmly established the relationship between *mda-9/syntenin* expression and melanoma metastasis [8, 45]. In the present study, we evaluated *mda-9/syntenin* as a host factor and defined whether host expression could influence tumor growth when B16 cells were implanted subcutaneously in *mda-9*^−/−^ mice and their WT littermates (C57BL/6). B16 cells abundantly express MDA-9/syntenin (data not shown, [46]. Overall, B16 cells grew faster in WT mice compared with *mda-9*^−/−^ mice. During the time course of the experiment (from day 3 to day 24) delayed tumor growth was observed in the *mda-9*^−/−^ *vs*. *mda-9* WT mice (Figure 1B). The tumor volumes from WT mice were ~2-fold greater than tumor volumes from *mda-9*^−/−^ mice at day 15 post-inoculation. By day 18 and 24, ~2.5-fold greater tumor volumes were found in WT mice as compared to *mda-9*^−/−^ mice. The average tumor weight was ~40% less in the *mda-9*^−/−^ group than in the WT group (Figure 1C). In total, these results suggest that *mda-9/syntenin* deficiency in the microenvironment negatively impacted tumor (melanoma) growth. H & E sections from WT mice indicated substantial pigmented cells in tumors, which were less apparent in the *mda-9*^−/−^ group (Figure 1D).

### *mda-9/syntenin* deficiency in the lungs of mice modulates B16 lung nodule growth

**Figure 2:**
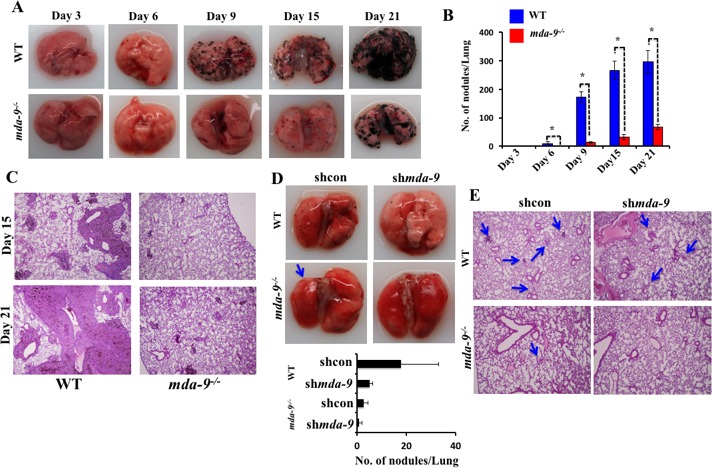
Lung metastasis development is inhibited in mice lacking *mda-9/syntenin* **A.** Formation of metastatic nodules in the lungs of WT and *mda-9* mice. A cohort of 15- age (6 weeks) and sex (male) matched wild type (C57BL/6) and *mda-9* mice were inoculated with B16 cells (1 × 10^5^) by intravenous injection to generate experimental lung metastases. Every 3^rd^ day, 3 mice from each group were sacrificed and lungs were collected, fixed with formalin and examined for nodules. Representative photographs of lungs with tumor metastases are presented. **B.** Graphical representation of average surface nodule number/mouse from each time point is presented. **C.** Photomicrographs of gross hematoxylin/eosin-stained lung sections are shown. **D.** In a separate study, a cohort of 6 animals from each mouse type (WT and *mda-9*) was inoculated with shcon or sh*mda-9* transfected B16 cells through tail-vein injection. After 6 days, lungs from each group were analyzed for lung nodule development. Representative photographs and the average number of nodules/mouse from each experimental group are shown in the upper and lower panel, respectively. **E.** Representative photomicrographs of gross lung hematoxylin/eosin stained lung sections are shown.

The lungs are the most common site for melanoma metastases [47]. Injection of B16 cells through the lateral tail vein results in pulmonary metastasis in C57BL/6 animals. To define a potential impact of *mda-9/syntenin* deficiency in the host lung on the development of metastatic nodules, B16 cells were injected into the tail vein of *mda-9*^−/−^ and WT mice. Lung metastases were evaluated by gross morphology of the lungs (Figure 2A), determining the number of metastatic nodules on the lung surface (Figure 2B), and lung morphology (presence of tumor and lung structure) (Figure 2C) on days 15 and 21 [48]. Overall, the WT group manifested enhanced pathology compared with *mda-9*^−/−^ mice, as indicated by an increased number of pigmented melanoma nodules in the lungs at each time point (Figure 2A). Visible lung nodules were first apparent between day 3 to day 6 after B16 injection in WT mice, while nodules only became apparent at day 6 in the *mda-9*^−/−^ mice, and dramatically fewer nodules were present at this time point (*mda-9*^−/−^: 0.33 ± 0.57 *vs*. WT: 9.33 ± 5.5). Similarly, at later time points (day 9, 15 and 21) the difference in lung nodule numbers was also significantly different between these two groups (Figure 2A and 2B). Additionally, the size of nodules was comparatively larger at all later time points (day 15 and 21, Figure 2C) in WT mice.

In a second experiment, *mda-9/syntenin* expression was downregulated in B16 cells using shRNA for *mda-9/syntenin* (sh*mda-9*), and these and control cells (shcon) were injected *via* tail vein into both WT and *mda-9*^−/−^ mice. After 6 days, mice were sacrificed and nodules were counted. As predicted, silencing of *mda-9/syntenin* in B16 cells significantly reduced their ability to form tumor nodules in WT mice (Figure 2D, WT: shcon *vs*. sh*mda-9* panel). As observed in our initial experiments using unmodified B16 cells (Figure 2A), fewer nodules were apparent in *mda-9*^−/−^ mice injected with B16 shcon (Figure 2D, shcon WT *vs*. shcon *mda-9*^−/−^ panel). In direct support of a role of *mda-9/syntenin* expression in the microenvironment impacting on tumor development, we did not observe any visible nodules in the *mda-9*^−/−^ mice that received *mda-9/syntenin* knockdown B16 (sh*mda-9*) cells (Figure 2D and 2E).

### Inflammatory and immunosuppressive cells are reduced in the lungs of *mda-*9 mice

**Figure 3:**
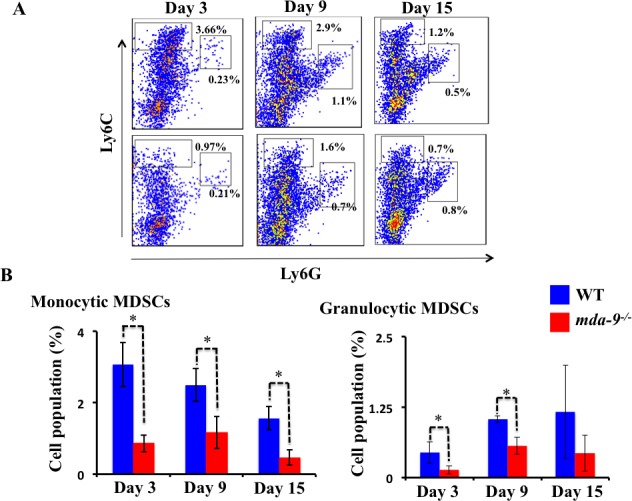
Infiltration of MDSCs is diminished in *mda-9*mice **A.** Accumulation of MDSCs in tumor bearing mice. A cohort of 12 animals from each experimental group received B16 cells (1 × 10^5^ cells/mouse) through intravenous injection. Three mice from each group at different time points were sacrificed, single-cell suspensions of tumor bearing lungs were prepared and analyzed for MDSCs accumulation by flow cytometry (experimental protocol provided in Methods and Materials). Representative histograms from each group are presented. **B.** Graphical presentation of average monocytic and granulocytic MDSCs (% of cells in the total population) from three animals at each time point ± S.D.

Since we observed a lower number of metastatic nodules in the lungs of *mda-9*^−/−^ mice as compared with their WT counterparts (Figure 2), we hypothesized that *mda-9/syntenin* expression in the tumor microenvironment might result in an alteration in tumor-supporting inflammation in *mda-9/syntenin*-deficient mice. To test this assumption, we first analyzed the expansion of MDSCs in the lungs of tumor-bearing WT and *mda-9*^−/−^ mice using flow cytometry. Following the establishment of metastatic B16 tumors, lungs from WT mice contained significantly higher numbers of both monocytic MDSCs (M-MDSCs, CD11b^+^Ly6C^high^Ly6G^−^) and granulocytic MDSCs (G-MDSCs, CD11b^+^Ly6C^low^Ly6G^+^) than those from *mda-9*^−/−^ mice (Figure 3A and 3B). The differences were significant for M-MDSCs, a prominent subtype for melanoma [49], at all time points analyzed in this study (day 3, 9 and 15). It is worth noting that at day 21, the accumulation of MDSCs (both subtypes) in *mda-9*^−/−^ mice were not significantly different from the wild type (Supplementary Figure S2). It is possible that the lack of *mda-9* expression in the microenvironment has a more significant impact on host-mediated anti-tumor activity at early stage of tumor progression and over time the tumor cells in the niche overcome the immune suppression and their growth is expanded. Consistent with this hypothesis, we observed nodule sizes (representing increased tumor cell number) were dramatically larger on day 21 compared with day 15 (Figure 2A) in *mda-9*^−/−^ mice.

**Figure 4:**
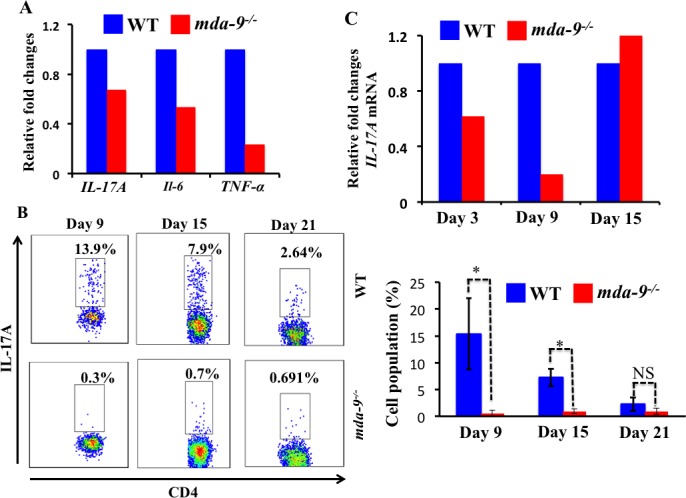
Knockout of *mda-9/syntenin* results in reduced *IL-17A* transcript expression and Th17 cell accumulation **A.** Real-time PCR was performed to determine the different cytokines (*IL-17A, IL-6* and *TNF-α)* at mRNA level in naive lungs, isolated from age- (8-weeks) and sex- (male) matched WT and *mda-9*−/− mice. Values were normalized with endogenous mouse *GAPDH*. **B.** Infiltration of CD4^+^Th17 cells in tumor bearing mice. A portion of lung cells from the experiment shown in Figure 3 was stained to identify IL-17A producing CD4 cells (CD4^+^Th17) cells. Representative histograms and average value are presented in the upper and lower panels, respectively. **C.** mRNA expression level for *IL-17A* in tumor bearing lung tissue isolated from tumor bearing mice at different time points (day 3, 9 and 15).

Interestingly, we observed several pro-inflammatory cytokines such as *IL-17A*, *IL-6* and *TNF-α* that were downregulated (at an mRNA level) in *mda-9*-deficient naïve lungs, suggesting a potential impairment of pro-tumorigenic responses to invading tumor cells ( Figure 4A). To determine the consequences of absence of *mda-9* in the lungs of mice, we next examined the infiltration of CD4^+^Th17 populations in the lungs of WT and *mda-9*^−/−^ mice in response to intravenous injected B16 cells. Flow cytometry analysis revealed lower numbers of CD4^+^ Th17 cells in the lung tumors from *mda-9*^−/−^ mice as compared to WT mice only at days 9 and 15 (total accumulation was gradually decreases as tumor progression occurred in both groups). Again, consistent with the MDSCs data presented in Supplementary Figure S2, CD4^+^ Th17 populations at day 21 were similar between WT and *mda-9*^−/−^ mice. To evaluate potential changes in the expression of the pro-inflammatory cytokine IL-17A in response to B16 cell colonization, qPCR was performed with RNA isolated from mouse lungs. At day 3, lower levels of IL-17A mRNA were evident in *mda-9*^−/−^ mice which was comparable to that of the naïve lungs (compare Figure 4A *vs* Figure 4C). At this time point, we did not find any difference in CD4^+^ Th17 cells (data not shown). A significant downregulation of *IL-17A* mRNA (Figure 4C) correlated with the CD4^+^ Th17 cells at day 9 (Figure 4B and Figure 4C). However, at day 15, we did not observe any differences of *IL-17A* mRNA in *mda-9*^−/−^ and WT groups. We did not explore the molecular mechanism in detail, but it is possible that other resident cells of the tumor microenvironment such as macrophages or T cells might produce IL-17A as a consequence of the presence of localized tumor cells. Overexpression of IL-17A might then facilitate the suppression of the host-mediated immune surveillance and allow the tumor cells to grow in *mda-9*^−/−^ mice, as evident in day 15 or later time points (Figure 2B). Further systematic studies are required to explore these phenomena, which are currently a focus in our laboratory.

### Deletion of *mda-9/syntenin* in *BRaf/Pten/Tyr-cre* mice delays tumor onset and inhibits formation of distant metastases

**Figure 5:**
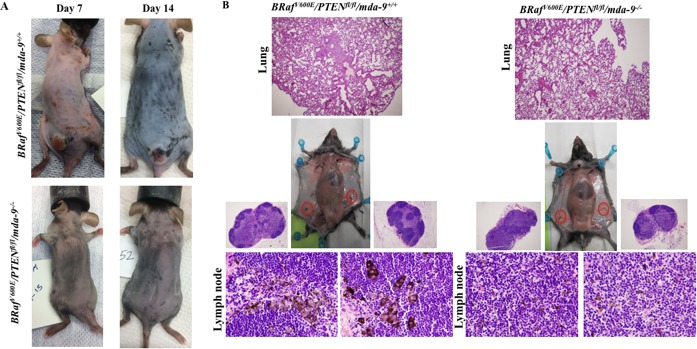
Lack of *mda-9/syntenin* delays tumor progression and lymph node and lung metastasis development in a transgenic mouse model of melanoma **A**. Comparison of *BRaf/PTEN/mda-9* (Upper panel) and *BRaf/PTEN/mda-9* (Lower panel) tumor progression in the early stage of tumor induction by tamoxifen (7 and 14 days after 4-HT induction). **B**. Metastasis to lung and inguinal lymph nodes (black color) is shown at 28 days after 4-HT injection. Representative H & E stained lung and lymph node sections are shown.

In the *BRaf^V600E^/Pten^fl/fl^/Tyr-cre^ER2^* mouse model, tamoxifen-mediated activation of *cre* results in activation of expression of the *Braf* oncogene and knockout of the *Pten* tumor suppressor gene specifically in melanocytes [50]. In the absence of tamoxifen, most of these mice display no discernible phenotype. However, topical or systemic administration of 4-hydroxytamoxifen (4-HT) results in the rapid development of melanomas that recapitulate the key pathophysiological features of human melanoma, including metastases in lymph nodes and lungs, with animals requiring euthanasia within 3-6 weeks. To comprehend the role of *mda-9/syntenin* in melanoma progression, we crossed *mda-9*^−/−^ mice with the *BRaf^V600E^/Pten^fl/fl^/Tyr-cre^ER2^* animals to develop mice that lack MDA-9/Syntenin expression. Systemic administration of 4-hydroxytamoxifen (4-HT) to parental *BRaf^V600E^/Pten^fl/fl^/Tyr-cre^ER2^* mice induced pigmented lesions within 7 to 10 days (Figure 5A). However, deletion of *mda-9/syntenin* (*BRaf^V600E^/Pten^fl/fl^/Tyr-cre^ER2^/mda-9*^−/−^) resulted in delayed tumor formation (Figure 5A). Additionally, we also observed less metastasis to regional lymph nodes and lungs in *BRaf^V600E^/Pten^fl/fl^/Tyr-cre^ER2^/mda*-9*^−/−^* (Figure 5B), which were assessed pathologically (presence of pigmented cells or lung morphology). These results provide further documentation of the role of *mda-9/syntenin* expression in the microenvironment in regulating primary tumor and metastasis formation in an autochthonous transgenic mouse model of melanoma containing an intact immune system.

## DISCUSSION

The primary direction of research on MDA-9/Syntenin (SDCBP) in cancer over the past two decades focused on defining its role in the context of tumor cells from different organ sites (7-11, 30,31,33,45,52,53) Although expression is significantly lower in normal *vs*. transformed cells, the MDA-9/Syntenin protein is ubiquitously expressed in the mouse (in both developing and adult stages [28, 51]) and in adult human organs, e.g., skin, liver, gall bladder, colon (www.Proteinatlas.org). Tamura *et al*. first demonstrated that knockout of MDA-9 (syntenin-1; SDCBP) in mice was not embryonic lethal and mice that developed did not show obvious abnormalities when grown in pathogen-free conditions [28]. The *mda-9*^−/−^ mouse model used in the present study was independently generated and characterized, corroborating the prior results that deletion of *mda-9/syntenin* in all tissues of the mouse is not lethal. In agreement with Tamura *et al.* [28], we also hypothesize that the lack of *mda-9/syntenin* in this animal is probably compensated by another variant of Syntenin, such as Syntenin-2, thereby preventing this knockout from being lethal.

MDA-9/Syntenin functions as a scaffold protein that interacts with a multitude of partner protein(s) and is involved in the generation or stabilization of active complexes, which play quintessential roles in multiple physiological activities [52, 53]. In this way *mda-9* can elicit various effects depending on its location in a cell and its binding partners. As indicated, previous studies have focused primarily on the role of *mda-9* in cancer cells without defining the role of this molecule in normal tissue and the microenvironment. We hypothesized that lack *of mda-9/syntenin* expression might impact significantly only when cells were challenged with exogenous insults, such as those resulting from implantation or injection of tumor cells. To validate this hypothesis, we examined the tumorigenic/metastatic behavior of B16 cancer cells when placed in contact with *mda-9/syntenin* deficient tissue in an *mda-9*^−/−^ mouse. As predicted, tumor growth and metastasis was suppressed in *mda-9*^−/−^ mice as compared with WT animals. Migration and seeding of cancer cells in the lung after tail vein injection depends on the interactions of tumor cells and the resident host cells, i.e., the microenvironment [54]. Many studies have shown that the microenvironment has the capacity to eliminate, minimize or enhance tumorigenesis [55, 56] depending on its composition and the stromal cell proportions or their activation states [34, 57].

Our study reveals that ablation of *mda-9/syntenin* impairs tumor-promoting inflammation and tumor-associated immunosuppression, characterized by fewer inflammatory and immunosuppressive cells, e.g., MDSCs, Th17 in the lungs with metastases. Malignant progression is often associated with inflammation and immunosuppression in the tumor sites [58]. The potential tumor-promoting role of MDSCs has been well documented in studies involving multiple animal models (reviewed by Quail *et al*, [55, 59] and clinical samples [60]. Pro-inflammatory IL-17A-producing Th17 cells, are often associated with tumors (reviewed Guery *et al*,[61]) with both good and bad prognoses. The tumor-promoting mechanism of Th17 cells is dependent on the production of IL-17A, a pro-inflammatory cytokine, that is known to promote tumor angiogenesis [62, 63], stimulate tumor cells to produce IL-6 [43], and in some murine models recruit MDSCs within tumors [44]. Additionally, Th17 cells may also exert immunosuppressive functions *via* direct inhibition of tumor-specific CD8^+^ T effector cells [64], and development of IL-17^+^Foxp3^+^ regulatory T cells [65]. The recruitment and expansion of various immune cells at least partially define the inflammatory status of the tumor microenvironment [66]. Although the precise mechanisms require further clarification, our results indicate that the basal levels of two major pro-inflammatory cytokines IL-6 and IL-17A are downregulated in the lungs from *mda-9*^−/−^ mice. IL-17A is predominantly produced by immune cells, including T cells, macrophages, dendritic cells (DC), natural killer cells, natural killer T cells, and γδ-T cells [67]. Tissue resident cells such as fibroblasts also modulate the local inflammatory conditions by producing various cytokines and chemokines [57]. Although context dependent, STAT-3 activation has been reported to be involved in IL-17 regulation [68, 69]. Considering that we have previously shown that MDA-9/Syntenin can activate STAT-3 in cancer cells [33], we postulate that lack of *mda-9/syntenin* in our *mda-9*^−/−^ mice may abrogate STAT-3-mediated IL-17 production. Precisely how MDA-9/Syntenin regulates IL-17 expression is currently an area of active investigation.

Although somewhat controversial, IL-17 is often considered to exert its' tumor-promoting effects in the context of inflammation [55]. More precisely, when IL-17 is exogenously produced in the microenvironment, tumor growth is enhanced as documented in studies with IL-17^−/−^ mice [43, 44, 70] or intra-tumoral injection of IL-17 siRNA [71]. Using B16 cells, Wang *et al* [43] demonstrated that IL-17A induced IL-6 production by tumor cells, which in turn enhanced tumor growth in a STAT-3-dependent manner. Additionally, this study also revealed strong CD^+^8 T cell accumulation and IFN -γ production in IL-17^−/−^ mice. Our *mda-9*^−/−^ mouse model, which has a lower level of *IL-17* mRNA compared to WT mice, also displayed reduced B16 tumor growth and lung metastases. It is possible that the lower expression of *IL-17* in *mda-9*^−/−^ mouse lungs impairs infiltration of MDSCs, an inflammatory cell population with immunosuppressive activity. Although we did not see complete regression of tumors (at the primary or metastatic sites), kinetics of tumor development and pathological state of tumor-bearing lungs correlated with the accumulation of these inflammatory and immunosuppressive cells. Finally, our hypothesis that *mda-9/syntenin* expression in stroma is also important in tumor and metastatic development is validated in a spontaneous melanoma metastasis model. Additional studies will be needed to further understand the cross-talk of tumor cells (either expressing MDA-9/Syntenin or not) within an *mda-9/syntenin* deficient tumor microenvironment, which will provide further insight into the multiple levels of involvement and functions of this gene in this complex process.

**Figure 6:**
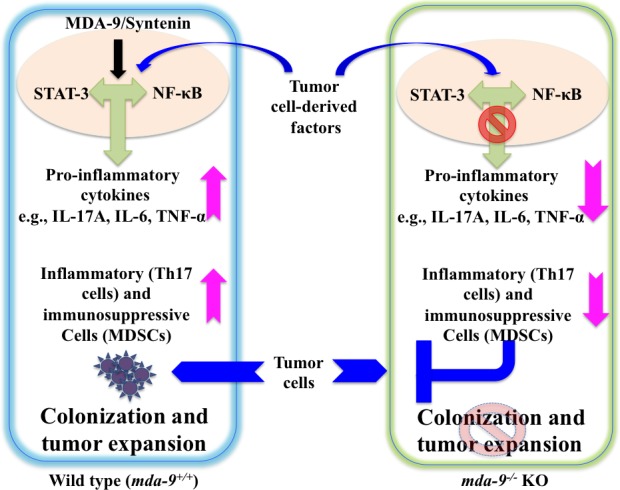
Model depicting the proposed role of host environmental *mda-9/syntenin* (*SDCBP*) expression in defining melanoma metastasis Tumor cell-derived factor(s) may trigger an autocrine/paracrine loop resulting in the production of pro-inflammatory cytokine(s) in the microenvironment through STAT-3/NF-κB pathways. This positive loop and accumulation of pro-inflammatory cytokines then recruit inflammatory and immunosuppressive cells (e.g., Th17 cells and MDSCs) to support and facilitate tumor outgrowth. We hypothesize that MDA-9/Syntenin plays a critical role in this early induction phase of the metastatic process by promoting an inflammatory and immunosuppressive environment, possibly *via* the STAT-3/NF-κB pathways. Absence of MDA-9/Syntenin negatively impacts on the inflammatory cascade and blocks the mobilization of inflammatory and immunosuppressive cells, which, as a consequence, leads to reduced tumor cell colonization and/or growth.

The present study is the first demonstration of the relevance of MDA-9/Syntenin expression in regulating inflammation and immunosuppression in the microenvironment thereby impacting tumor growth and metastasis. A schematic model of the role of MDA-9/Syntenin in the tumor cell and microenvironment on metastasis is shown in Figure 6. Taking into account our previous research (7-11, 31,45,52,53) the present study also supports the newer concept that MDA-9/Syntenin is a key protein product influencing metastasis by regulating both tumor cells and the microenvironment. Accordingly, this protein is an attractive therapeutic target for inhibiting metastasis. In principle, *mda-9/syntenin* could be targeted for inhibition genetically, using shRNA or siRNA delivered by nanoparticles or other approaches. For this strategy to be successful it would be necessary for the inhibitory RNAs to be delivered to metastatic cells in diverse and sometimes difficult to access regions of the body and to efficiently reduce expression to a level that would be therapeutically efficacious. Since a key mechanism by which MDA-9/Syntenin regulates cellular phenotypes is through protein-protein interactions through its PDZ domains, identifying small molecule inhibitors that specifically target the PDZ domains thereby disrupting the interactions and altering down stream signaling critical for activity would be of significant value. We are currently exploring both of these strategies to capitalize on the potential importance of *mda-9/syntenin* as a regulator of cancer invasion and metastasis in melanoma and other cancers. Finally, the *mda-9*^−/−^ mouse models we have created will pave the way in comprehending the role of the microenvironment in the development and therapy of melanoma and potentially additional cancers.

## MATERIALS AND METHODS

### Mouse model generation and breeding strategies

#### *mda-9/syntenin* knockout mice

To investigate the physiological role of *mda-9/syntenin* in metastasis development we generated a line of global *mda-9/syntenin* knockout mice (*mda-9*^−/−^), starting with a line of *mda-9* “knockout first” mice (*Sdcbp*^tm1a(KOMP)Wtsi^) obtained from the Knockout Mouse Project (KOMP) Repository [72], which were bred first to β-actin-FLPe mice (JAX stock #005703) to generate the conditional knockout (CKO) allele in which exon 3 is floxed. These mice were then bred to EIIa-cre mice (JAX stock #003724) to delete exon 3, which results in a translational frame-shift, leaving only 17 residues of the 299-residue MDA-9/Syntenin protein. We confirmed the absence of MDA-9/Syntenin protein in multiple tissues (lung shown) from mice homozygous for the knockout allele as well as the original “knockout first” allele, and retention of expression in mice with the floxed allele (Figure 1A).

#### *Compound Braf/Pten/Tyr-cremda-9* mice

The BRaf*^V600E^*/Pten*^fl/fl^*/Tyr-cre*^ER2^* tamoxifen-inducible genetically engineered mouse model of metastatic melanoma (51) accurately recapitulates the full process of tumor initiation, progression and metastasis. This model was crossed with *mda-9*^−/−^ mice to develop BRaf*^V600E^*/Pten*^fl/fl^*/Tyr-cre*^ER2^/mda-9*^−/−^ mice. Production of the BRaf*^V600E^*/Pten*^fl/fl^*/Tyr-cre*^ER2^* and BRaf*^V600E^*/Pten*^fl/fl^*/Tyr-cre*^ER2^/mda-9*^−/−^ mice were particularly challenging because of the large number of genetically modified alleles being bred into each mouse. To facilitate efficient production of these mice, we generated sub-colonies of optimal breeders. For example, to generate *BRaf^V600E^/Pten^fl/fl^/Tyr-cre^ER2^* mice of the desired genotype (heterozygous for a modified Braf allele that expresses WT Braf until cre is expressed, and is then converted to the mutant BRaf*^V600E^* allele; homozygous for a Pten floxed allele, and hemizygous for the Tyr-cre*^ER2^* transgene), we generated sub-colonies of: i) *BRaf^V600E^/Pten^fl/fl^/Tyr-cre^ER2^* and ii) *Braf^fl/fl^* / *Pten^fl/fl^* (*cre*-) mice. When mice of these two genotypes were interbred, 50% of the offspring were of the experimental genotype, while the remaining 50% were cre- controls. Similarly, we also generated the following parental sub-colonies to develop the *mda-9*^−/−^ mice: iii) *Braf^WT/WT^* / *Pten^fl/fl^* / *Tyr-cre^ER2^* / *mda-9*^−/−^ and iv) *Braf^fl/fl^* / *Pten^fl/fl^* / *mda-9*^−/−^ (*cre-*) mice.

### Western blotting analysis

Freshly isolated organs (e.g., Lung) were minced with sterile scalpels and incubated with cell lysis buffer for 30 minutes on ice followed by homogenization using a glass homogenizer. All steps were performed on ice. Protein lysates were collected after centrifugation. For western blotting, we used MDA-9/syntenin (1:50, Cat. No. sc-100336) and β-actin (1:2000, Cat No. A-1978) antibodies from Santa Cruz Biotechnology ( Dallas, TX) and Sigma-Aldrich (St. Louis, MO), respectively.

### RNA extraction, cDNA preparation, and real-time PCR

Total RNA was extracted from different mouse organs as indicated using QIAGEN miRNAeasy Mini Kit (QIAGEN). After confirming the quality of RNA, ABI cDNA Synthesis Kit (Applied Biosystems, Carlsbad, CA) was used to prepare cDNA. Real time PCR was performed using an ABI ViiA7 fast real-time PCR system and TaqMan gene expression assays according to the manufacturer's protocol. All relevant probes (mouse specific Taqman probe for IL-17A, IL-6, TNF-α, GAPDH) were purchased from Applied Biosystems (Carlsbad, CA).

### Xenograft and experimental metastasis

For xenograft experiments, 2 × 10^5^ B16 cells were subcutaneously injected in the right dorsal flank of mice as previously described [73]. Tumor volume was measured twice weekly with a caliper and calculated using formulas *π*/6 × larger diameter × (smaller diameter)^2^ [74]. Experimental lung metastasis was performed as previously described [73]. Briefly, B16 cells (1 × 10^5^ cells per mouse) suspended in 100 μL PBS (without Ca^2+^ and Mg^2+^) were injected intravenously to generate pulmonary metastases. Based on the experimental design, mice were sacrificed at different time points.

### Routine pathological staining

Tumors or organs were fixed in formalin. Samples were then embedded in paraffin, sectioned, and haematoxylin/eosin stained by the Virginia Commonwealth University Anatomic Pathology Laboratory.

### Flow cytometry analysis

Lungs from tumor-bearing mice were collected in cold RPMI and single cell suspensions were prepared as described previously [73, 75]. Equal numbers of cells were re-suspended in FACS buffer (PBS containing 0.1% BSA and 0.04% EDTA-Na_2_). After surface staining with the appropriate antibody [(e.g., for MDSCs, PE-CD11b^+^ (MI/70), PerCP/cy5.5-Ly6C (HK1.4) and APC-Ly6G (1A8); for Th17 cells, APC-CD4 (GK1.5) and PerCP/Cy5.5-IL-17A (TC11-18H10.1)] as previously described (75), flow cytometry analysis was performed using a BD FACSCalibur flow cytometer. Data were analyzed using FlowJo software (Tree star, Ashland, OR). Antibodies were purchased from BioLegend (San Diego, CA).

### Statistical analysis

Data are shown as the average ± S.D. Unpaired two-tailed Student t-tests were conducted for calculating the “*p*” value between two groups. A “*p*” value <0.05 was considered statistically significant.

## SUPPLEMENTARY MATERIALS AND FIGURES


