# Supplementary material for: Knockout of MDA-9/Syntenin (SDCBP) expression in the microenvironment dampens tumor-supporting inflammation and inhibits melanoma metastasis
Source: Oncotarget. 2016 Jun 21;7(30):46848–61. doi: 10.18632/oncotarget.10040 (PMC5216907; doi:10.18632/oncotarget.10040)
Supplement: Supplementary file 1 [file oncotarget-07-46848-s001.pdf]

Knockout of MDA-9/Syntenin (SDCBP) expression in the microenvironment dampens tumor-supporting inflammation and inhibits melanoma metastasis

Supplementary Material

Supplementary Fig

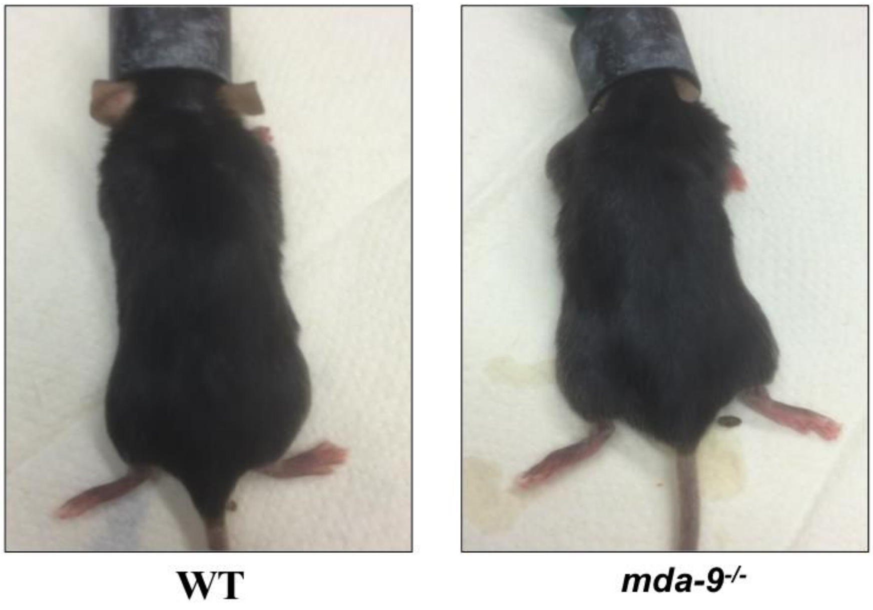

Supplementary Fig. S1. Representative photographs of sex- (male) and age- (8 weeks) matched Wild type and *mda-9*$^{-/-}$ mice.

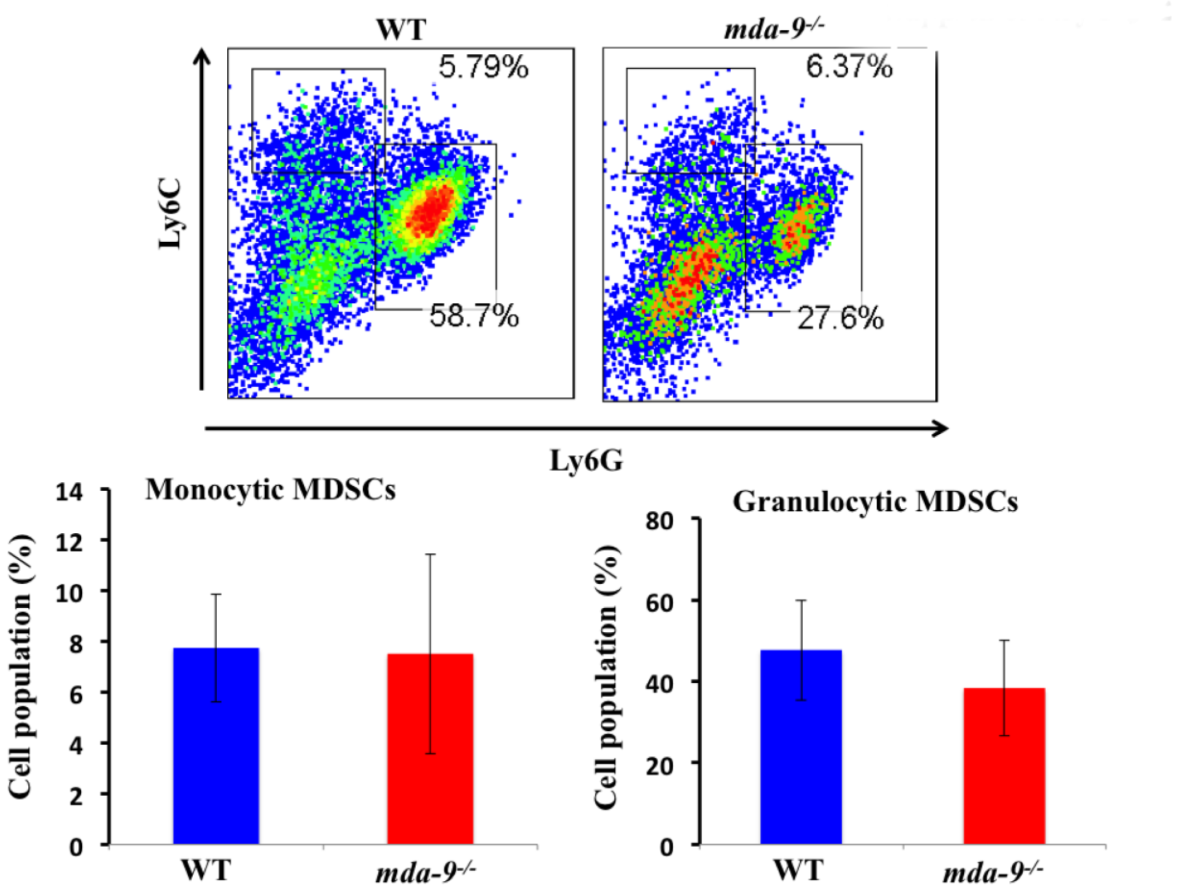

Supplementary Fig. S2. Accumulation of MDSCs in tumor bearing mice. A. Representative histograms for MDSCs from day 21 group (experimental protocol provided in Methods and Materials) are presented. B. Graphical presentation of average monocytic and granulocytic MDSCs (% of cells in the total population) from three animals at day 21 time point ± S.D.
